# Prevalence of Pulp Stones in the Population of Rajasthan: A Cross-Sectional Study in a Tertiary Care Hospital

**DOI:** 10.7759/cureus.51623

**Published:** 2024-01-03

**Authors:** Pravin Kumar, Arunkumar Duraisamy, Arun Patnana, Karishma Pathak, Vinay Chugh

**Affiliations:** 1 Conservative Dentistry and Endodontics, Department of Dentistry, All India Institute of Medical Sciences, Jodhpur, IND; 2 Pedodontics and Preventive Dentistry, Department of Dentistry, All India Institute of Medical Sciences, Rajkot, IND; 3 Orthodontics and Dentofacial Orthopedics, Department of Dentistry, All India Institute of Medical Sciences, Jodhpur, IND

**Keywords:** dental calcifications, root canal therapy, rajasthan population, intraoral periapical radiographs, pulp stones

## Abstract

Introduction

This study aimed to use radiography to determine the prevalence of pulp stones in the population of Rajasthan and to evaluate the relationship between pulp stones and tooth status, type, age, and gender.

Methods

The radiograph data record files collected from the Department of Dentistry, All India Institute of Medical Sciences, Jodhpur, Rajasthan, from September 2018 to October 2019, had a total of 9918 diagnostic quality intraoral periapical radiographs. One examiner examined all the radiographs to identify pulp stones and associated factors. Pearson chi-square test of significance was used for statistical analysis.

Results

On screening, a total of 889 intraoral periapical radiographs were found to have pulp stones. The presence of pulp stones was significantly higher in mandibular molars (68%) and was more common in the age group of 31-45 years (37%), followed by 13-29 years (35%). Maximum of pulp stones were of attached type (64%) than free pulp stones.

Conclusion

The prevalence of pulp stones in the population of Rajasthan studied is 8.9%, which is much lower than the reported prevalence in the literature. Pulp stones are predominantly attached and found significantly more often in mandibular molars in the age group of 31-44 years.

## Introduction

Pulp stones are calcified masses seen in the dental pulp tissue of healthy and damaged teeth. These pulp stones are more common in the coronal section of the pulp canal space than in the radicular segment [1,2]. These pulp stones can either exist freely inside the pulp tissue or be adherent (connected to the wall of the pulp space but not totally encompassed by dentine) or embedded in the dentin, posing a challenge during endodontic treatment. The size of the pulp stones ranges from minute particles to enormous masses that can totally obliterate the pulp area on a radiograph. The number of pulp stones discovered varies widely, from 0 to 12 or more in certain teeth [3,4]. They may occur in any type of tooth, although they are most common in permanent molars [2,5-7].

The types of pulp stones, or denticles, can be divided into three structural categories: diffuse/amorphous, true, and false. True pulp stones are made of dentin and include distinguishable dentinal tubules bordered by odontoblasts whereas false pulp stones are made of collagen fibers, dying or dead cells, or concentric layers of mineralized tissue produced around blood thrombi [6]. A diffuse or amorphous pulp stone is found around blood vessels and has a more asymmetrical shape than a false pulp stone. False pulp stones arise when degenerating pulp cells finally mineralize, while true pulp stones are thought to form as a result of epithelial-mesenchymal interactions. In addition, trauma, tooth transplantation, pulpal circulation, orthodontic tooth movement, and older age are other causes [2,8]. It has been associated with systemic illnesses such as cardiovascular disease, renal disease, and systemic sclerosis drugs, as well as hereditary predispositions, such as dentin dysplasia, dentinogenesis imperfecta, and Van der Woude syndrome [9-11]. Their occurrence is sometimes considered idiopathic.

Pulpal calcifications can occur at any age, and a recent analysis estimated the incidence across different populations' teeth to range from 2.1% to 27.8% [12]. Various radiographic studies found that the incidence of pulp stones is between 20% and 25% [7,13,14], although histologic examinations revealed a higher prevalence [2,15]. These histological investigations, typically regarded as the gold standard to analyze any tissue sample for composition, invariably indicate a larger number of calcifications. The frequency of pulp stones was observed to be 9.09% in the northern Indian central Punjabi population and 17.9% in Andhra Pradesh, India [16,17]. On orthopantomogram, the prevalence of pulp canal calcification in Karnataka was observed to be 6% [18]. The study of the prevalence of pulp stones may also aid in understanding pulp physiology and the rate of disease progression and pulpal reactions to the infection in specific population groups. Reporting the present study data may also aid researchers around India to compare with other population groups. However, no such prevalence studies of pulp stones were done in the population of Rajasthan. Hence, the study aimed to use intraoral periapical (IOPA) radiographs to determine the prevalence of pulp stones in the population of Rajasthan and to evaluate the relationship between pulp stones and tooth status, type, age, and gender.

## Materials and methods

Study design and recruitment of participants

This cross-sectional (retrospective) study was carried out using the data of radiographs in the Department of Dentistry, All India Institute of Medical Sciences, Jodhpur, after receiving clearance from the Institute Ethical Committee (IEC Reference No.: AIIMS/IEC/2019-20/1014). All digital IOPA radiographs taken during routine investigation were included in the study, as the pulp stone would be more clearly visible in IOPA radiographs.

Inclusion and exclusion criteria

All IOPA radiographs taken during the 14 months since 2018 for regular out-patient examination reported to dental OPD service, Department of Dentistry, and radiographs that were diagnostically acceptable were included in this study, whereas radiographs that were of poor quality, elongated/shortened, faulty radiographs (cone-cut), or not satisfied for radiographic diagnosis were excluded.

Pulp stone analysis

**Figure 1:**
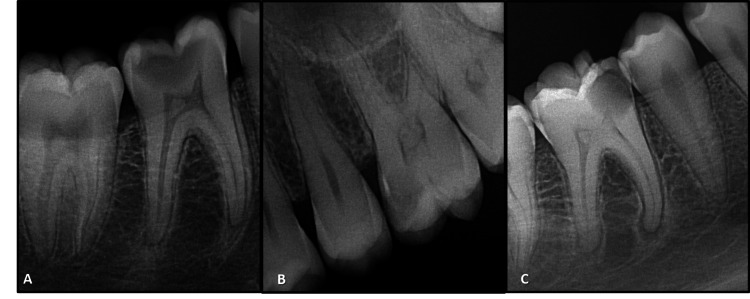
Types of pulp stones. (A) Free pulp stone, (B) attached pulp stone, and (C) both.

The radiographic (IOPA) data records were retrieved and analyzed. All radiographs were read by one examiner in a dimmed room, focusing attention on pulp stones. IOPA radiographs of good diagnostic quality were included in the present study, whereas those with radiographic errors such as improper horizontal or vertical angulations, and inadequate exposure causing scoring difficulties were excluded. A total of 9918 IOPA radiographs were screened in the radiographic data records of the Department of Dentistry to identify the pulp stones in the teeth. Demographic data, presence of pulp stone, and radiographic appearance were recorded and categorized for analysis purposes. A tooth was scored as having a pulp stone when a definite radiopaque mass was observed in the pulp space. A radiopaque mass fully surrounded by pulpal tissue was identified as a free pulp stone, whereas an attached pulp stone was identified as partially fused with dentin (Figure 1). A thorough case record form used for the study contained the data. By rereading a random sample of 10% of the total radiographs previously reviewed, the examiner's reliability was determined.

Statistical analysis

The data were statistically analyzed using SPSS version 21 software (IBM Corp., Armonk, NY) to report the prevalence of pulp stones. We examined variations in pulp stone percentages by sex, age, tooth type, and dental arch. Intra-examiner reliability was evaluated using Cohen's kappa statistics and it was reported to be 0.95. Pearson chi-square test of significance was used for statistical analysis.

## Results

**Figure 2:**
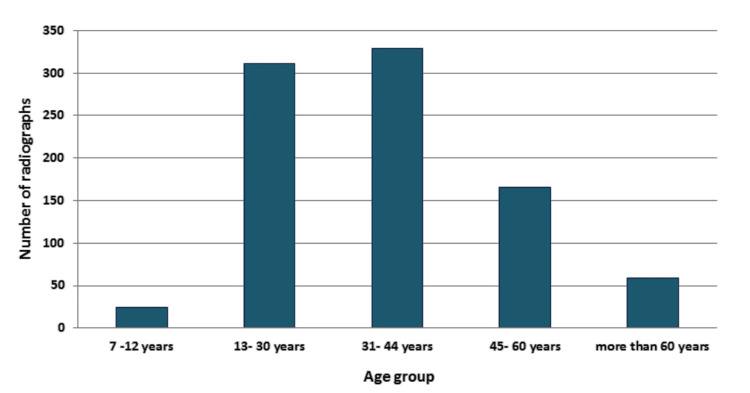
Distribution of pulp stones according to age groups.

Among the available radiographic data records, 5306 were of male and 4612 were of female subjects, while 742, 3517, 3955, 1644, and 960 radiographs belonged to different age groups, i.e., 7-12 years, 13-30 years, 31-44 years, 45- 60 years, and more than 60 years, respectively. A total of 889 radiographs were identified with pulp stones from the screened dental records (889/9918). The total prevalence of pulp stones in the given population was 8.9%. Intra-examiner reliability was found to be 0.95. Maximum pulp stones were observed in the age group of 31-44 years (37%), followed by the 13 to 30 years age group (35%). A total of 437 (49.1%) male and 452 (50.8%) female subjects exhibited pulp stones (Figure 2).

**Figure 3:**
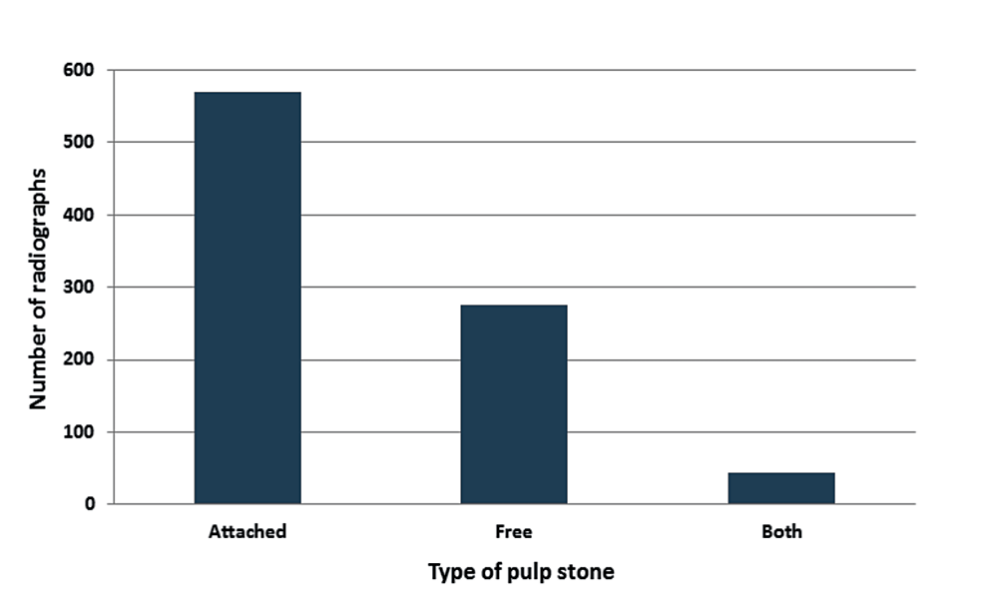
Type of pulp stones according to the location in the pulp chamber.

The type of pulp stones according to the location in the pulp chamber is presented in Figure 3. Maximum pulp stones were observed as attached (64%) to the walls of the pulp chamber, followed by free pulp stones (31%).

**Figure 4:**
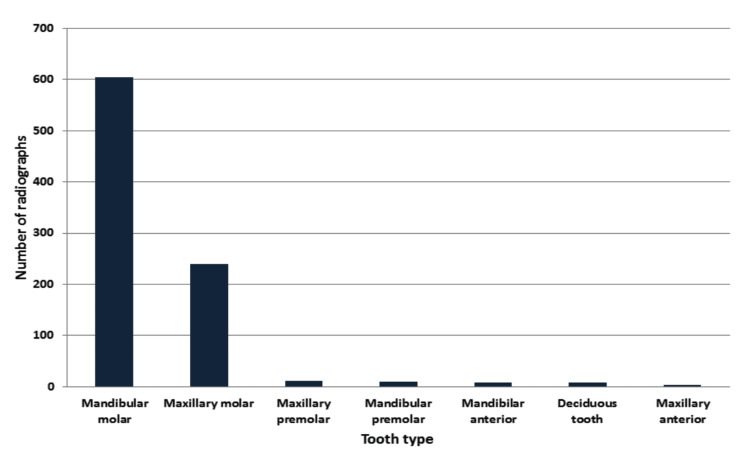
Distribution of pulp stones in different teeth.

The distribution of pulp stones in different teeth is presented in Figure 4. Mandibular molars (68%) showed a higher percentage of pulp stones, followed by the maxillary molars (27%).

## Discussion

When pulp stones are greater than 200 µm in diameter, they can be detected using dental radiography. A radiographic investigation is the only noninvasive method accessible for routine investigations, even though the true occurrence of a pulp stone is probably greater in histologic analysis of teeth. However, there were no significant differences in the diagnosis of pulpal calcification between bitewing and periapical radiographs [13,14]. As bitewing radiographs do not reveal the entire radicular pulp, we chose to examine IOPA radiographs obtained from the previous database for the presence of pulp stones.

A study of 814 adult Jordanians reported that pulp stones were identified in 22% of the teeth and 51% of the patient's radiographs [13]. While Ranjitkar et al. studied the prevalence of pulp stones in 217 dental students from Australia, they discovered that 10% of the teeth and 46% of the subjects had them. A study conducted by Kannan et al. on 361 patients in Malaysia found that 15.7% of the teeth examined and 44.9% of the patients had pulp stones [19].

According to the data in the literature, 50% of all teeth will have one or more pulp calcifications [20]. The current study, on the other hand, found a prevalence of 8.9%, which was lower than previously published literature. Variations in the prevalence rate might be attributed to the populations investigated as well as methodological differences in the research designs. The frequency of pulp stones in the Indian population ranged from 9% to 18% in north Indian central Punjab and south Indian Andhra Pradesh [2,13,21]. There has been no study published to assess the prevalence of pulp stones in the Indian population as a whole. As a result, the data presented in this study on the population of Rajasthan may add to the literature on the prevalence of pulp stones in the western Indian population.

We found that the age group of seven to 12 years old had the lowest frequency of pulp stones, while the age group of 31 to 44 years old had the highest incidence (37%). This complements the findings of Hamasha and Darwazeh by demonstrating that there is no real correlation between pulp stones and advancing age [13]. The frequency of pulp stones in females was almost equal to that of males. Contrary to previous studies, the association was not very strong.

Molars had a higher prevalence of pulp stones, which was similar to previous published data [2,7,13,21]. According to Hamasha and Darwazeh, molars have a greater blood flow to the pulp tissues, resulting in increased calcium precipitation. However, the presence of pulp stones was more common in mandibular molars than in maxillary molars, which was consistent with the study by Baghdady et al. [7].

Both carious and restored teeth may develop pulp stones as a result of persistent pulpal irritation. Pulpal calcifications can result from the pulp-dentinal complex's defense response to caries and microleakage around restorations. In terms of mechanics, pulp stone development may be similar to tertiary dentin formation near inflamed odontoblasts. In a study by Sener et al. [22], pulp calcifications resulted from long-term irritants, and pulp stone incidence was higher in teeth that were restored or carious, as well as teeth that had both caries and restorations. Our study revealed a considerable incidence of pulp stones in teeth that were otherwise undamaged, in contrast to earlier findings. However, pulp stones have been found in immature teeth and primary teeth, indicating that pulp disease is not the sole etiology of pulp stone development. Periodontal disease was attributed to the production of pulp stones by Rubach and Mitchell, but neither pulp stones nor dispersed calcifications were associated with bone loss [23]. Recently, calcifying nanoparticles have also been proposed as an etiologic cause for pulp stones [11]. Therefore, future research may focus on how all other factors contribute to pulp stone formation in teeth that are periodontally compromised teeth.

Our investigation revealed pulp stones in primary teeth, which is consistent with a study by Mello-Moura et al. (2017) [24]. Pulp stones were much more common in people with dilacerations, impactions, taurodontism, and enamel pearls, according to study findings by Hamasha and Darwazeh [13]. On the other hand, our investigation revealed no association between dental defects and pulp stone incidence. In the current investigation, only a few participants had pulp stones in more than one tooth. The calcifications seen varied in size from tiny opacities to large radiopaque masses that completely obstructed the pulp chamber.

Some researchers reported prevalence based on the number of patients and teeth, but others solely represented rates based on the number of teeth. We estimated prevalence based on the number of IOPA radiographs and completely visible teeth counted on radiographs in this study.

The current clinical consensus is that pulp stones have limited clinical significance other than the possibility of producing challenges during endodontic therapy, such as obstructing canal identification and negotiation. In forensic dentistry, radiographic matching of pulp stone shapes, together with other characteristics documented in dental records, can help identify deceased people or victims of crime.

The limitations of this study stem from the nature of cross-sectional analysis, which may not provide definite information about the cause-and-effect relationship. Other variables that may induce pulpal irritation, such as tooth conditions (caries) and regressive changes of tooth and/or periodontium, were not recorded in detail. Further investigation is needed to understand the etiological variables that contribute to pulp stone production, as well as other factors that may promote pulpal irritation, such as tooth surface loss or periodontal diseases. The size, form, position, and quantity of pulp stones on radiographs can be utilized as an additional characteristic for forensic identification; nevertheless, these findings need more research.

## Conclusions

The prevalence of pulp stones in the population of Rajasthan studied is 8.9%, which is lower than the reported prevalence in the literature. Pulp stones are predominantly attached, and found significantly more often in mandibular molars in the age group of 31-44 years. Endodontists and clinicians may consider the prevalence rate of pulp stones in the population of Rajasthan for successful management of decayed teeth.
